# Recurrent Metastatic Malignant Wolffian Tumour: A Rare and Aggressive Clinical Course

**DOI:** 10.7759/cureus.106551

**Published:** 2026-04-06

**Authors:** Junzhe Zhao, Yen Ching Yeo, Yash B Boricha, Kazila Bhutia, Maili Qi

**Affiliations:** 1 Obstetrics and Gynaecology, Duke-NUS Medical School, Singapore, SGP; 2 Department of Pathology and Laboratory Medicine, KK Women's and Children's Hospital, Singapore, SGP; 3 Department of Obstetrics and Gynaecology, KK Women's and Children's Hospital, Singapore, SGP; 4 Department of Urogynaecology, KK Women’s and Children’s Hospital, Singapore, SGP; 5 Department of Gynaecological Oncology, KK Women’s and Children’s Hospital, Singapore, SGP

**Keywords:** adjuvant chemotherapy, fatwo, immunohistochemistry, recurrence, wolffian tumour

## Abstract

Wolffian tumours (female adnexal tumours of probable Wolffian origin, FATWO) are rare mesonephric duct-derived neoplasms with variable malignant potential and challenging diagnosis. A 68-year-old woman underwent complete resection for a left tubo-ovarian mass and was diagnosed with a localised Wolffian tumour with malignant potential. After 22 months of surveillance, she re-presented with malignant ascites and extensive peritoneal dissemination showing anaplastic transformation and underwent cytoreductive surgery. Adjuvant chemotherapy was poorly tolerated and ineffective, with rapid progression despite treatment, leading to best supportive care. This case highlights the diagnostic complexity and potential aggressive behaviour of malignant Wolffian tumours, the limited efficacy and tolerability of chemotherapy in advanced disease, and the need for early integration of palliative care when disease becomes refractory.

## Introduction

The Wolffian duct is an embryonic structure present in both genders during early development. In females, it largely regresses, leaving vestigial remnants that may give rise to Wolffian tumours, also known as female adnexal tumours of probable Wolffian origin (FATWO) [1]. In males, Wolffian remnants may form tumours in the paratesticular region, epididymis, or seminal vesicles. Although traditionally considered a tumour of low malignant potential, an increasing number of case reports and small series have demonstrated that approximately 10-20% Wolffian tumours may behave aggressively, with local recurrence, peritoneal dissemination, and distant metastases [1,2]. Since the 1970s, fewer than 130 cases have been reported worldwide [1].

The pathophysiology and molecular drivers of malignant progression remain poorly understood because of the rarity of the disease. Histologically, these tumours also pose diagnostic challenges because of their overlap with other gynaecological neoplasms such as mesonephric-like carcinoma or sex cord stromal tumours [2], making immunohistochemistry (IHC) and clinico-pathological correlation essential for diagnosis. Emerging reports have described molecular alterations involving *KRAS*, *PIK3CA*, and *TP53* pathways, although robust genomic characterisation remains limited.

We present a rare case of malignant Wolffian tumour with recurrence and rapid metastasis following an initial 22-month disease-free interval, complicated by malignant pleural effusion, chemotherapy intolerance, and progressive clinical deterioration. It highlights the aggressive biology of this rare disease and the challenges of its diagnosis, surveillance, and systemic management.

## Case presentation

A 68-year-old Malay woman with two previous normal vaginal deliveries presented with postmenopausal bleeding in December 2021. Her medical history included metachronous Stage IIA invasive ductal carcinoma of the left breast (ER-, PR+, HER2-) in complete remission following surgery, chemotherapy, radiotherapy and hormonal treatment between 2011 and 2022.

**Figure 1:**
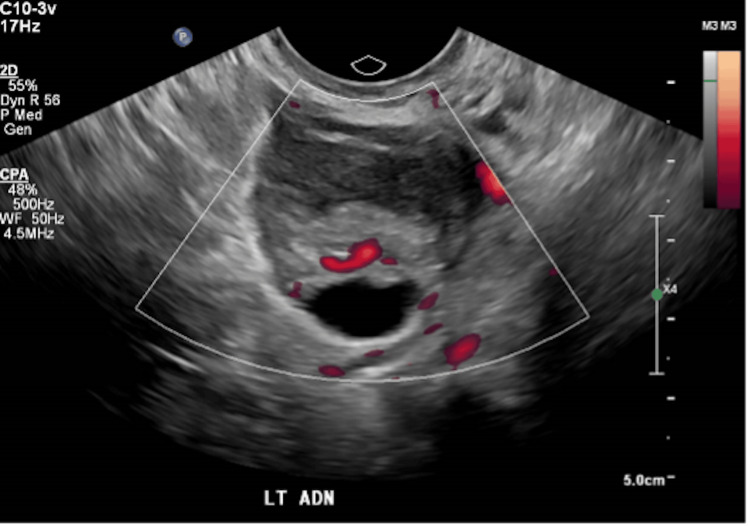
Ultrasound pelvis showed a 3.7 x 3.0 x 2.8 cm tubo-ovarian complex, with convoluted tubular cystic structure with low-level echoes and a segment with a thick vascular wall.

Initial pelvic ultrasound (Figure 1) demonstrated a suspicious left tubo-ovarian complex (O-RADS US = 4). She underwent dilatation and curettage, which revealed atrophic endometrium and a benign endometrial polyp. Tumour markers at diagnosis included CA-125 at 12.8 U/mL and AFP at 32 ng/mL. In May 2022, she underwent a total laparoscopic hysterectomy with bilateral salpingo-oophorectomy, pelvic and para-aortic lymphadenectomy, and infracolic omentectomy. Intraoperative findings revealed a 5 cm left adnexal solid-cystic mass with dense adhesions to the sigmoid colon and bladder, partially obliterating the pouch of Douglas. No ascites or peritoneal metastases were observed, and complete resection was achieved. Frozen section reported sex cord tumour favouring granulosa cell tumour.

**Figure 2:**
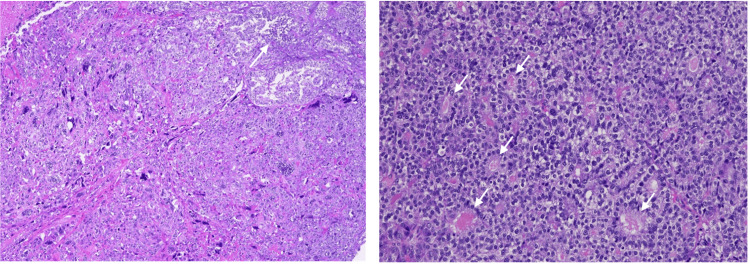
Histopathology of the initial resected tumour. Left: The tumour is composed largely of small- to medium-sized rounded glands interspersed with solid-looking tubules, nests, and sheets. Areas of necrosis (arrow) are noted (40x). Right: The tumour cells are monotonous, cuboidal and low columnar, with rounded and hyperchromatic nuclei showing granular chromatin and small nucleoli. Nuclear grooves are not present. Cytoplasm varies from clear to amphophilic. Many of the glands (arrows) contain dense eosinophilic secretions. Scattered mitoses are seen (100x).

The final histopathological examination (Figure 2) demonstrated a tumour composed of small- to medium-sized rounded glands interspersed with solid tubules, nests, and sheets. Tumour cells were monotonous, cuboidal to low columnar, with hyperchromatic nuclei, granular chromatin, and small nucleoli. Cytoplasm ranged from clear to amphophilic, with dense eosinophilic luminal secretions. Necrosis and variable mitotic activity were present. The origin of the tumour was difficult to ascertain, but the bulk of the tumour appeared to be localised within the left adnexal soft tissue, with involvement of the tubal fimbria and serosal surfaces. The tumour abutted the left ovary but with no definite involvement of the ovary. Definitive subtyping of the lesion was difficult, but based on the morphology and IHC profile (Table 1), a diagnosis of Wolffian tumour, likely malignant, was favoured. Following multidisciplinary discussion (MDT), given complete resection and lack of evidence supporting adjuvant therapy, the patient opted for active surveillance.

**Table 1:** Immunohistochemistry at the patient's initial diagnosis and recurrence.

Marker	Initial diagnosis (May 2022)	Recurrent tumour (April 2024)
ER	Negative	Negative
PR	Negative	Negative
c-KIT	Negative	Negative
MOC-31	Positive (diffuse)	Not reported
CD99	Positive (diffuse)	Not reported
CK7	Positive (patchy)	Positive
EMA	Positive (patchy)	Positive
WT-1	Positive (focal)	Negative
Inhibin	Positive (focal)	Positive (focal)
Calretinin	Negative	Not reported
GATA3	Negative	Positive (diffuse in anaplastic area)
CD10	Negative	Positive (diffuse)
AE1/3	Not reported	Positive
p53	Not reported	Aberrant (overexpression)
β-hCG	Not reported	Positive (in multinucleated cells)
CK20	Not reported	Negative
PAX8	Negative	Positive (focal)
Vimentin	Positive (Focal weak)	Not reported

**Figure 3:**
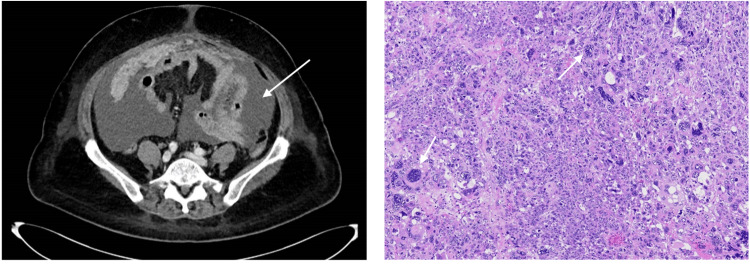
Recurrent tumour in April 2024. Left: CT shows gross ascites (arrow) with extensive peritoneal disease and omental caking. Right: Histopathology of the recurrent tumour shows scattered glands, resembling the initial tumour, intimately associated with solid sheets of malignant cells with scattered bizarre and multinucleated giant cells (arrows) indicative of malignant transformation (40x).

The patient remained disease-free for 22 months following surgery, with stable tumour markers during surveillance and unremarkable imaging in December 2023. Just three months later, however, she developed malignant ascites with marked biochemical progression, including CA-125 1,178 U/mL, AFP 5 ng/mL, and β-hCG 4,123 IU/L (Table 2). Ascitic fluid cytology and omental biopsy revealed metastatic adenocarcinoma with new diffuse positivity for GATA3, PAX8, CD10, and aberrant p53 (overexpression). Imaging demonstrated extensive peritoneal disease (Figure 3). Laboratory assessment during recurrence also demonstrated hypoalbuminaemia and thrombocytosis, consistent with high disease burden and declining physiological reserve (Table 2).

**Table 2:** Selected laboratory findings across the clinical course. Units: CA-125: U/mL; AFP: ng/mL; β-hCG: IU/L; Hb: g/dL; Platelets: ×10^9^/L; albumin: g/L. Abraxane/Carbo: nab-paclitaxel with carboplatin.

Timepoint	CA-125	AFP	β-hCG	Hb	Platelets	Albumin	Key notes
May 2022 (Initial diagnosis)	12.8	32	<2.3	11.4	319	Normal	Initial diagnosis
Mar 2024 (Recurrence)	1,178	5	4,123	10.1	689	17	Malignant peritoneal disease
Jul 2024 (Pleural effusion)	295.3	5	2,388	11.0	499	33	Malignant pleural effusion
Aug 2024 (Post Abraxane/Carbo)	150.4	5	727.4	10.6	637	26	Grade 4 neutropenia

Given the discrepancy in histology from the original tumour, the differential diagnoses included tumour recurrence versus de novo peritoneal carcinoma. Considering that she had a long disease-free interval and optimal debulking deemed achievable on the CT scan, cytoreductive surgery was performed in April 2024, achieving no gross residual disease. 

On histology (Figure 3), the recurrent tumour was composed mainly of solid islands and nests of malignant cells with enlarged nuclei, conspicuous nucleoli and eosinophilic cytoplasm. Scattered multinucleated and bizarre tumour cells were noted, with focal glandular differentiation. In the better differentiated areas with gland formation, the histological features appeared similar to the original tumour seen in the initial resection, with a similar IHC profile (Table 1). The recurrent tumour showed a more anaplastic and higher-grade morphology compared with the primary lesion, consistent with malignant transformation. The findings supported recurrence of a malignant Wolffian tumour.

Adjuvant chemotherapy with carboplatin and paclitaxel was initiated in June 2024 but was complicated by severe anaphylactic shock to paclitaxel, myocardial ischaemia, and acute lactic acidosis. Subsequent treatment with carboplatin and nab-paclitaxel was poorly tolerated, resulting in Grade 4 neutropenia and gastrointestinal toxicity with rapid performance status deterioration.

Between June and November 2024, rapid disease progression occurred, with recurrent malignant pleural effusions requiring repeated drainage. Imaging revealed extensive retroperitoneal, supraclavicular, and cardiophrenic lymphadenopathy, new peritoneal deposits, and pleural-based masses. Given aggressive progression and chemotherapy intolerance, the patient and family opted for best supportive care with palliative team involvement.

The final illness phase was characterised by severe, persistent back pain, anorexia, profound fatigue, and progressive respiratory symptoms due to bilateral pleural effusions. The patient died in December 2024, 30 months after initial diagnosis.

## Discussion

The present case demonstrates the diagnostic complexity and aggressive potential of malignant Wolffian tumours. In the early stages, symptoms are variable and non-specific. Definitive diagnosis relies heavily on IHC, which is critical for distinguishing Wolffian tumours from other adnexal malignancies, including high-grade serous carcinoma, granulosa cell tumour, and mesonephric-like carcinoma [2,3]. Key positive diagnostic markers include pancytokeratin, CK7, alpha-inhibin, CD10, and GATA3; key negative markers include TTF1, CK20, PAX8, and EMA in most cases [4,5]. The recurrent tumour demonstrated focal positivity for PAX8 and EMA, highlighting the histopathological heterogeneity of Wolffian tumours.

At recurrence, the markedly elevated serum β-hCG (4,123 IU/L) and β-hCG positivity restricted to multinucleated giant cells (Table 1) raise the differential diagnosis of true trophoblastic differentiation or a germ cell component. However, aberrant or ectopic β-hCG expression with syncytiotrophoblast-like multinucleated cells has been described across a range of poorly differentiated/high-grade non-trophoblastic carcinomas [6,7], and does not by itself establish a germ cell tumour or gestational trophoblastic neoplasia. In our case, the β-hCG-positive cells were sparse and occurred in the anaplastic component, supporting this as a high-grade transformation phenomenon rather than a discrete trophoblastic neoplasm.

In this patient, an aberrant p53 profile in the recurrence is atypical for classic Wolffian tumours, as the largest molecular series has not identified *TP53* as a recurrent driver alteration [4]. Nevertheless, *TP53*-pathway alteration may reasonably be acquired during malignant transformation or dedifferentiation, including in Wolffian tumours [8]. Accordingly, we interpret the aberrant p53 as a feature of high-grade transformation; paired molecular profiling of primary and recurrent lesions would be valuable in future cases to confirm clonality and define actionable alterations. The marked change in immunophenotype and acquisition of aberrant p53 expression at recurrence may reflect clonal evolution and tumour dedifferentiation during disease progression.

Malignant Wolffian tumours demonstrate aggressive biological behaviour with rapid progression, extensive metastases, and significant anaplastic transformation despite initial low-stage presentation and prolonged disease-free intervals [9,10]. Metastasis typically occurs within the peritoneal cavity but can extend to pleural spaces and lymph nodes, as demonstrated in this case. Awareness of these metastatic patterns is crucial for surveillance planning.

Complete surgical resection remains the standard treatment for Wolffian tumours. However, no established standard of care exists for adjuvant therapy or management of recurrent disease. Most patients with completely resected early-stage Wolffian tumours do not receive adjuvant chemotherapy due to limited evidence of outcome improvement and recognition that most tumours are indolent [11]. Decisions for adjuvant therapy are often made on an individual basis for cases with aggressive histopathological features, such as high mitotic index, nuclear pleomorphism, hypercellularity, and extratumoural extension [12]. For recurrent or metastatic disease, chemotherapy is often initiated, particularly with peritoneal involvement and high-grade transformation. Carboplatin and paclitaxel, extrapolated from ovarian cancer protocols, are the most frequently used regimen [12]. However, response rates are highly variable, and severe toxicities are common.

Molecular profiling and targeted therapy approaches represent emerging frontiers in Wolffian tumour management. Recent studies have identified recurrent genetic alterations such as mutations in *KRAS*, *PIK3CA*, and *CTNNB1* genes, with frequent loss of heterozygosity at chromosome 22q [13]. These molecular insights may provide therapeutic targets, particularly given the limited efficacy of conventional chemotherapy. Imatinib mesylate, a tyrosine kinase inhibitor, has shown promise in isolated case reports, with one patient achieving a complete response and another maintaining stable disease [14]. Comprehensive molecular profiling remains limited, and prospective studies of targeted therapies are lacking due to the rarity of these tumours. Emerging targeted therapies, including PI3K/AKT/mTOR pathway inhibitors and PARP inhibitors, may offer therapeutic options based on the molecular profile of individual tumours [15]. Routine molecular testing may therefore be considered for patients with malignant Wolffian tumours. Collaboration through international registries and multi-institutional studies will be essential to advance our understanding of the molecular biology and optimal therapeutic approaches for these rare tumours.

## Conclusions

This case underscores the importance of vigilant follow-up, early palliative care involvement, and patient-centred decision-making in rare tumours with uncertain behaviour. The rapid progression and severe symptom burden highlight the critical role of clear prognostic communication and end-of-life planning. Given the limited literature on malignant Wolffian tumours, there remains no consensus on optimal surveillance strategies or systemic therapy, particularly once disseminated disease or anaplastic transformation develops. Until higher-quality data emerge, management should be individualised within a multidisciplinary team, balancing the potential benefit of cytoreductive approaches against toxicity and the patient’s goals. Comprehensive case reporting and multi-centre registries may be essential to strengthen prognostication and develop pragmatic care pathways for this rare malignancy.
